# Inhibition of 14-3-3 Proteins Alters Neural Oscillations in Mice

**DOI:** 10.3389/fncir.2021.647856

**Published:** 2021-03-12

**Authors:** Zachary B. Jones, Jiajing Zhang, Yuying Wu, Yi Zhou

**Affiliations:** Department of Biomedical Sciences, Florida State University College of Medicine, Tallahassee, FL, United States

**Keywords:** 14-3-3 proteins, neural oscillations, theta band, gamma band, schizophrenia, local field potentials

## Abstract

Accumulating evidence suggests that schizophrenia is a disorder of the brain’s communication, a result of functional and structural dysconnectivities. Patients with schizophrenia exhibit irregular neuronal circuit and network activity, but the causes and consequences of such activity remain largely unknown. Inhibition of 14-3-3 proteins in the mouse brain leads to the expression of multiple schizophrenia endophenotypes. Here we investigated how 14-3-3 inhibition alters neuronal network activity in the mouse hippocampus (HPC) and prefrontal cortex (PFC), key brain regions implicated in schizophrenia pathophysiology. We implanted monopolar recording electrodes in these two regions to record local field potentials both at rest and during a cognitive task. Through our assessment of band power, coherence, and phase-amplitude coupling, we found that neural oscillations in the theta and gamma frequency ranges were altered as a result of 14-3-3 dysfunction. Utilizing transgenic and viral mouse models to assess the effects of chronic and acute 14-3-3 inhibition on oscillatory activities, respectively, we observed several fundamental similarities and differences between the two models. We localized viral mediated 14-3-3 protein inhibition to either the HPC or PFC, allowing us to assess the individual contributions of each region to the observed changes in neural oscillations. These findings identify a novel role of 14-3-3 proteins in neural oscillations that may have implications for our understanding of schizophrenia neurobiology.

## Introduction

Schizophrenia is a chronic and disabling psychiatric disorder that affects approximately 3 million Americans, and the economic burden of schizophrenia is tremendous (Cloutier et al., 2016). Given that schizophrenia symptomology includes deficits in various cognitive domains, and as neural oscillations are a fundamental mechanism of cognition (Basar et al., 2001; Buzsaki and Draguhn, 2004), it has been hypothesized that the schizophrenic brain would exhibit dysfunctional oscillatory activity. Indeed, clinical and preclinical studies have provided evidence for dysfunctional network activity in schizophrenia, including abnormalities in low and high frequency oscillations, coupling between these oscillations, and connectivity between brain regions (Friston, 2002; Uhlhaas, 2013; Ribolsi et al., 2014; Sheffield and Barch, 2016; Hunt et al., 2017; Kang et al., 2018; Marissal et al., 2018; Bygrave et al., 2019). Nonetheless, much work remains to be done to determine the consequences of such disturbances and elucidate the mechanisms by which these disturbances come about.

Our lab has established the 14-3-3 function knockout (FKO) mouse model as a powerful tool to study neuropsychiatric behavioral endophenotypes and the pathophysiological basis of schizophrenia (Foote et al., 2015). These FKO mice transgenically express yellow fluorescent protein (YFP)-fused difopein (dimeric-fourteen-three-three peptide inhibitor) to disrupt the endogenous functions of 14-3-3 proteins in the brain using a neuronal-specific Thy-1 promoter. Difopein inhibits all seven mammalian isoforms of 14-3-3 proteins which constitute an evolutionarily conserved family of proteins that are highly expressed in the brain (Berg et al., 2003; Qiao et al., 2014). 14-3-3 proteins exist as homo- and heterodimers, with each monomer made up of nine antiparallel α-helices that form an amphipathic groove for binding ligands with phosphoserine/phosphothreonine motifs (Fu et al., 2000). They are known to interact with hundreds of proteins to regulate diverse neurobiological processes including neuronal development, apoptosis, and synaptic transmission (Skoulakis and Davis, 1998; Zhang and Zhou, 2018). 14-3-3 protein dysfunction has been implicated in a number of neurological diseases such as Parkinson’s disease, Alzheimer’s disease, bipolar disorder, and schizophrenia (Foote and Zhou, 2012; Kaplan et al., 2017; McFerrin et al., 2017; Fan et al., 2019; Gu et al., 2020).

We previously reported that our FKO mice, with high difopein transgene expression in the prefrontal cortex (PFC) and hippocampus (HPC), displayed behavioral and cognitive deficits that correspond to core schizophrenia endophenotypes. Treatment with antipsychotic drugs attenuated some of these deficits (Foote et al., 2015). Further, FKO mice displayed defects in synaptic physiology and function which correspond to schizophrenia-associated electrophysiological impairments (Qiao et al., 2014). The goal of this study is to build upon these findings by examining the role of 14-3-3 proteins in neural oscillations and determining whether inhibition of 14-3-3 proteins in the mouse brain recapitulates the dysfunctional network activity seen in patients with schizophrenia.

## Materials and Methods

### Animals

Generation of transgenic 14-3-3 FKO mice was previously described (Qiao et al., 2014). Briefly, these mice express YFP fused difopein (dimeric fourteen-three-three peptide inhibitor) using the Thy-1 promoter. Positive founder line mice were backcrossed to wild-type (WT) C57BL/6 mice for at least eight generations. Male and female FKO mice and WT littermates were group housed in single-sex, mixed-genotype cages on a 12:12 h light-dark cycle prior to experiments. Mice were 3–6 months old at the time of recording. All animal procedures were carried out in accordance with the guidelines for the Care and Use of Laboratory Animals of Florida State University and approved by the Florida State University Animal Care and Use Committee.

### Electrode Implantation and Recording

Custom made monopolar electrodes were constructed from 200 μm polyimide-coated stainless steel wire (Plastics One) soldered to gold connector pins (A–M Systems). They were implanted in the PFC (RC: +2.5 mm, ML: ±1.0 mm, DV: −1.5 to −2.0 mm brain surface, relative to bregma) and dorsal CA1 of the HPC (RC: −2.1 mm, ML: ±2.0 mm, DV: −1.4 mm brain surface, relative to bregma) using a stereotaxic frame (Kopf stereotaxic instruments). A ground/reference electrode was implanted in the cerebellum (RC: −6.0 mm, ML 0.0 mm, DV: −2.0 mm brain surface, relative to bregma). Up to five anchoring screws (J. I. Morris) were distributed around the skull, and the entire assembly was secured in place with dental cement (Stoelting Co.). After surgery, mice were individually housed for 1 week prior to recording.

Local field potentials (LFPs) were sampled at a rate of 1 kHz in the OpenEphys GUI (Siegle et al., 2017) with an RHD2000 USB interface board and RHD2132 16-channel amplifier board with a wire adaptor for custom electrode wiring (Intan). Video tracking was collected with an overhead mounted Flea3 camera (FLIR) at 30 frames per second. Timestamps were synchronized with LFPs in the OpenEphys GUI via TTL outputs.

### Behavior

#### Open Field Activity

Mice were placed into a square open field arena (Med Associates Open Field Arena, 43.2 cm L × 43.2 cm W × 30.5 cm H, with IR photobeam sensors) and their general activity was assessed for 30 min using Med Associates Activity Monitor software.

#### Y-Maze

The testing apparatus was constructed from opaque white acrylic and consisted of three arms (12” L × 3” W × 6” H) angled 120° apart in the shape of a “Y”. Visual cues were placed around the testing room. During testing, the mouse was placed on the center of the maze and allowed to freely explore for 10 min while activity was recorded. Video files were analyzed to determine the order of arm entries, total number of arm entries (T), and total number of complete alternations (Alt). A complete alternation was scored when the mouse consecutively entered all three different arms, e.g., B-A-C. The proportion of spontaneous alternations was calculated as Alt/(T-2). Mice that completed less than 12 arm entries over the 10 min period were excluded from analysis.

### Drug Administration

Intraperitoneal injections of clozapine (2 mg/kg, Tocris), haloperidol (0.4 mg/kg, Sigma-Aldrich) or saline (0.9% NaCl) were delivered 30 min prior to recording. Mice were randomly assigned to receive either clozapine or haloperidol to achieve a balanced design with respect to genotype and sex.

### Viruses

Yellow fluorescent protein-difopein containing adeno-associated virus (AAV) was constructed and produced by the Obio Technology (Shanghai) Co., Ltd. The cDNA encoding YFP-difopein was subcloned into the rAAV vector, pAOV-CaMKIIα-YFP-difopein (Graham et al., 2019). AAV-CaMKIIα-YFP purchased from the UNC Vector core was used as a negative control. These viruses (AAV serotype 2/9) were then produced using the triple transfection method in HEK 293 cells and AAV titers were determined by real-time PCR.

For virus injections, mice were deeply anesthetized with intraperitoneal injection of ketamine (70 mg/kg)/xylazine (10 mg/kg). A 33-gauge needle was positioned in the PFC (RC: +2.5 mm, ML: ±1.0 mm, DV: −1.5 to −2.0 mm brain surface, relative to bregma) or dorsal CA1 HPC (RC: −2.1 mm, ML: ±2.0 mm, DV: −1.4 mm brain surface, relative to bregma) using a stereotaxic frame (Kopf stereotaxic instruments). Viruses (0.5 μL) were infused slowly over 5 min into the targets with a 10 μL Hamilton syringe. Five minutes after infusion, the needle was slowly retracted. After injection, mice were individually housed for 2 weeks to allow for maximum virus expression prior to open field testing and electrode implantation.

### Histology

Mice were anesthetized and transcardially perfused with 4% paraformaldehyde in 0.1 M phosphate buffer, pH 7.4 (PBS). After an overnight postfixation in the same fixative at 4°C, the brains were then cut into 100 μm coronal sections on a vibratome (Leica Microsystems). Brain sections were stained with DAPI (1:1000, Invitrogen) for 10 min prior to mounting with Vectashield to retard fluorescence fading and were imaged on a Keyence BZ-X700 fluorescence microscope using a 2X objective.

### Experimental Design

To promote active exploration, Y-maze recordings were performed prior to resting-state recordings. For each recording session, mice were allowed to habituate to the experiment room in their home cage for at least 30 min. Following habituation, mice were gently placed in the Y-maze apparatus. After 10 min in the maze, mice were returned to their home cage and placed in a custom-built sound and light-attenuating chamber. Following a 5-min period of habituation to this new environment, resting-state LFPs were recorded for 10 min. All recordings were done inside a Faraday cage to minimize electrical noise. The number of mice per group (*n*) is indicated for each experiment. Recordings were excluded from analysis under the following conditions: ≥10% bodyweight loss at the end of recovery period, excessive noise in recordings, incorrect targeting of AAV injection or electrode implant, or insufficient alternations during Y-maze.

### Data Processing and Statistical Analysis

OpenEphys data files were read and analyzed in MATLAB (Release 2020a, Mathworks). A 1–100 Hz butterworth bandpass filter was applied to raw data with the *butter* function. Data artifacts were removed with the EEGLAB toolbox *pop_rejcont* function (Delorme and Makeig, 2004). A 60 Hz notch filter was applied to remove line noise using the *iirnotch* function. 1–100 Hz power spectra were calculated using the *pwelch* function (Welch’s power spectral density estimate, 2 s Hamming window, 50% overlap). Theta (4–10 Hz) and gamma (30–100 Hz) power were calculated using the *bandpower* function. Absolute band power in these predefined frequency ranges was divided by total band power across all frequencies (1–100 Hz) to calculate relative band power. Coherence was calculated using the *mscohere* function (10 s Hamming window, 50% overlap). Phase-amplitude coupling was quantified via the modulation index (Tort et al., 2010). For both coherence and phase-amplitude coupling, a distribution of 200 surrogate values was generated for each recording pair by randomly shuffling data points within one of the recordings. The 95th percentile of the distribution was used as a statistical threshold, and any coherence or modulation index values less than this threshold were set to zero. Granger causality was calculated with the Multivariate Granger Causality (MVGC) toolbox (Barnett and Seth, 2014).

All data are presented as mean ± standard error of the mean (SEM) and were assessed by either *t*-tests or ANOVAs with Tukey-Kramer HSD correction. *P*-values were adjusted for multiple comparisons with the Benjamini and Hochberg false discovery rate. A value of *p* < 0.05 was considered statistically significant. All statistical analyses were performed in MATLAB using the *t-test2*, *anovan*, *multcompare*, *mafdr*, and *corrcoef* functions.

## Results

We assessed neural oscillations via recorded LFPs, a measure of the summed electrical activity of large neuronal populations (Buzsaki et al., 2012; Pesaran et al., 2018). From these LFPs, we derived measures of power, coherence, and phase-amplitude coupling to quantify group differences. First, as abnormal EEG power, particularly in the theta (4–10 Hz) and gamma (30–100 Hz) frequency ranges, is characteristic of patients with schizophrenia and preclinical schizophrenia models (Moran and Hong, 2011; Sun et al., 2011; Gandal et al., 2012; Tatard-Leitman et al., 2015; Uhlhaas and Singer, 2015; McNally and McCarley, 2016; Mukherjee et al., 2019), we focused on these two frequency bands for our analyses. Second, as reduced global functional connectivity is a general feature of schizophrenia (Friston, 2002; Uhlhaas, 2013; Ribolsi et al., 2014), and the degree of functional connectivity is state-dependent (Mathalon and Sohal, 2015), we use coherence as a measure of connectivity between brain regions both at rest and during a cognitive task (Bowyer, 2016). Third, as phase-amplitude coupling of theta and gamma oscillations is recognized as an important modulator of memory and cognition (Tort et al., 2008, 2009; Lisman and Jensen, 2013; Colgin, 2015; Tamura et al., 2017; Nandi et al., 2019), and dysfunction in the neural circuits supporting this coupling in schizophrenia may be responsible for the disordered thoughts and deficits in learning and memory observed in patients, we used the modulation index to measure both local and inter-regional theta-gamma coupling (Moran and Hong, 2011; Mathalon and Sohal, 2015; Barr et al., 2017; Nandi et al., 2019). Together, these three measures of power, coherence, and phase-amplitude coupling allow us to assess how 14-3-3 inhibition alters neural oscillations in awake, behaving mice.

### Neural Oscillations in WT and 14-3-3 FKO Mice

**FIGURE 1:**
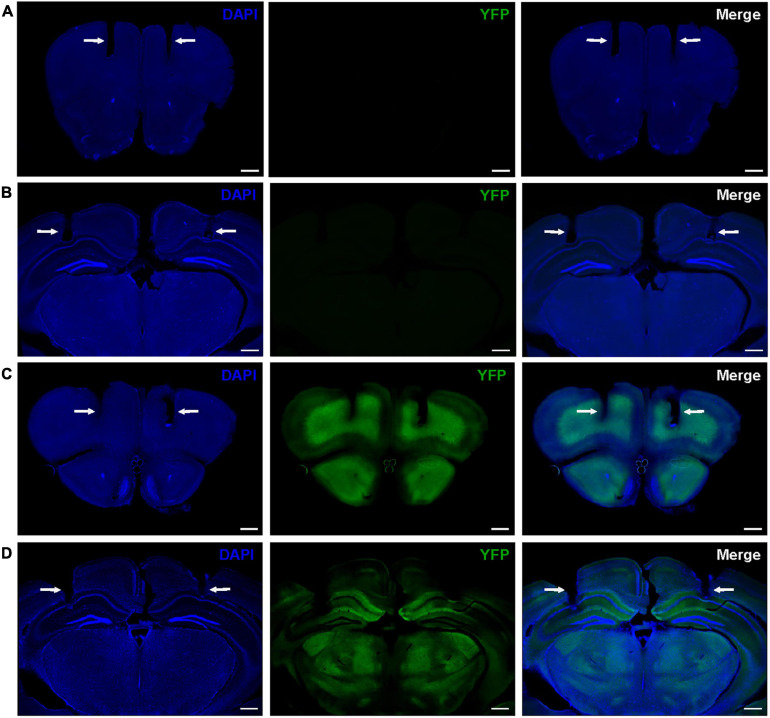
Representative histology showing locations of bilateral electrode tracks in the PFC **(A)** and HPC **(B)** of WT mice and the PFC **(C)**, and HPC **(D)** of 14-3-3 FKO mice, as indicated by white arrows. High expression of YFP-fused difopein transgene can be seen in 14-3-3 FKO mice **(C,D)**. Scale bar = 500 μm.

In this study, we focused on neural oscillations in the PFC and HPC for two reasons. First, these two regions have particularly high difopein expression in the FKO mouse line that exhibits schizophrenia-like behaviors (Foote et al., 2015). Second, both regions are critically involved in cognitive function (Preston and Eichenbaum, 2013; Shin and Jadhav, 2016). We bilaterally implanted chronic recording electrodes in the medial PFC and dorsal CA1 HPC of WT mice (Figures 1A,B) and 14-3-3 FKO littermates (Figures 1C,D) to determine how neural oscillations in varying behavioral states may be altered by 14-3-3 dysfunction. As no significant sex differences were observed (data not shown), data from male and female mice were pooled together for the subsequent analyses. In addition, no significant differences between bilaterally implanted electrodes were observed (data not shown), so data from left and right electrodes were averaged together for analysis.

#### Resting-State Oscillations

**FIGURE 2:**
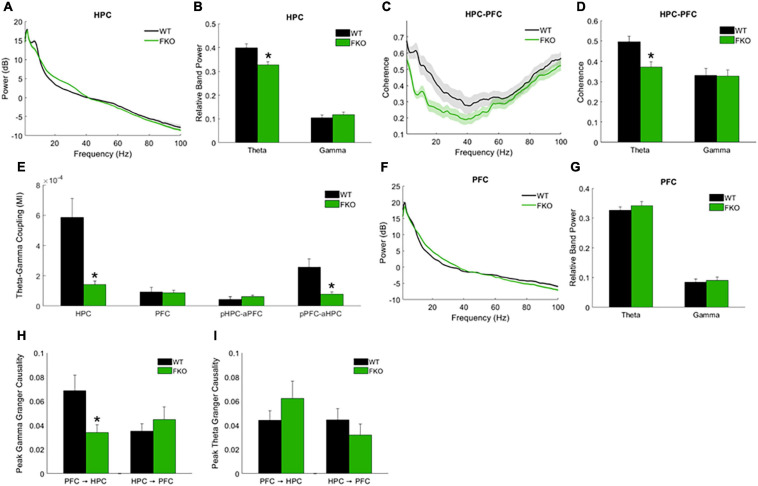
Resting-state neural oscillations in WT (*n* = 16) and 14-3-3 FKO mice (*n* = 19). HPC power spectra **(A)** and band power **(B)** measurements show that theta power is decreased in FKO mice. FKO mice exhibit decreased theta coherence between the HPC and PFC **(C,D)**. Theta-gamma coupling within the HPC and inter-regional coupling between PFC phase and HPC amplitude is decreased in FKO mice **(E)**, while coupling between HPC phase and PFC amplitude is not statistically different between FKO and WT mice. There are no statistically signifcant differences in the PFC theta or gamma power **(F,G)**. In the gamma frequency range, FKO oscillations exhibit reduced granger causality in the PFC to HPC direction, but not the reverse **(H)**. There are no differences in theta granger causality between WT and FKO oscillations in either direction **(I)**. Statistical significance denoted as *p < 0.05. MI, modulation index. pHPC, HPC phase. aPFC, PFC amplitude. pPFC, PFC phase. aHPC, HPC amplitude.

Resting-state network activity was assessed via spontaneous neural oscillation recordings from mice in their home cages (Supplementary Figure 1). The power of theta oscillations in the HPC (HPC theta power) of 14-3-3 FKO mice was significantly lower than that of their WT littermates (*p* = 0.0143, Figures 2A,B). Theta coherence between the HPC and PFC, thought to be important for effective long-range communication in the brain, was also significantly decreased in the FKO mice (*p* = 0.0198, Figures 2C,D). Moreover, theta-gamma couplings (Supplementary Figure 2), including both local coupling within the HPC (*p* = 6.72e-4) and inter-regional coupling between the PFC phase and HPC amplitude (*p* = 8.98e-4), were impaired in 14-3-3 FKO mice (Figure 2E). Interestingly, we did not observe any statistically significant differences between WT and FKO mice in PFC theta or gamma power (Figures 2F,G) or theta-gamma coupling within the PFC (Figure 2E). Further, coupling between the HPC phase and PFC amplitude (Figure 2E) was not significantly different between the FKO and WT mice. This highlights the region-specificity of neural oscillatory defects related to 14-3-3 dysfunction and suggests that communication between the PFC and HPC is asymmetrically disrupted in the FKO mice (Nandi et al., 2019; Jiang et al., 2020). In support of this view, further analysis revealed that granger causality, a measure of directed functional connectivity (Barnett and Seth, 2014), in the gamma frequency range was reduced in FKO mice compared to WT mice in the PFC to HPC direction (*p* = 0.0136), but not vice-versa (Figure 2H). There was no significant difference in theta granger causality between WT and FKO mice (Figure 2I). Together, these results demonstrate that functional 14-3-3 proteins are important for maintaining basal coordinated network activity in adult mice.

We previously reported that antipsychotic drug administration in 14-3-3 FKO mice attenuates specific schizophrenia-associated behavioral endophenotypes such as novelty-induced hyperlocomotion (Foote et al., 2015). While this behavior in mice does not directly correspond to a behavior seen in patients with schizophrenia, it has been recognized as a useful correlate of the positive symptoms of schizophrenia, i.e., an increase in abnormal behavior that is generally responsive to antipsychotics (Jones et al., 2011). We did not observe any statistically significant effects of these drugs on neural oscillations in either WT (Supplementary Table 1) or FKO mice (Supplementary Table 2). This is perhaps not surprising, as it is known that neither typical antipsychotics such as haloperidol nor atypical antipsychotics such as clozapine are effective in treating the cognitive symptoms of schizophrenia (Furth et al., 2013). As dopamine receptors are a primary target for these drugs, we may thus infer that the abnormal neural oscillations observed in FKO mice are not acutely dependent on dopamine signaling.

#### Task-Related Oscillations

**FIGURE 3:**
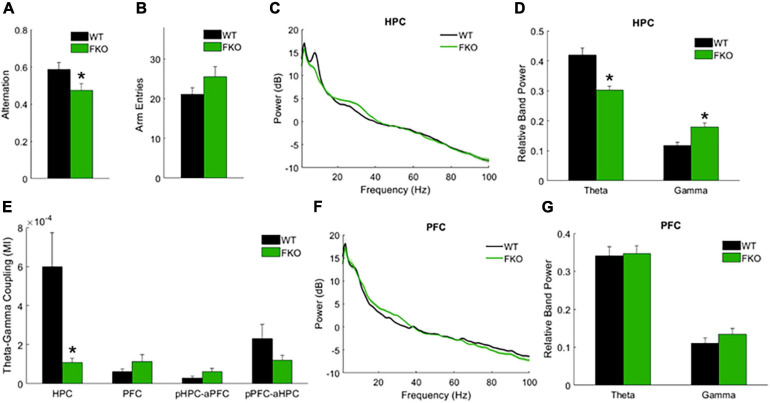
Task-related neural oscillations in WT (*n* = 12) and 14-3-3 FKO mice (*n* = 14). Y-maze spontaneous alternations were significantly decreased in FKO mice **(A)**, indicative of spatial reference memory impairment. There is no significant difference between WT and FKO mice in the number of arm entries during the Y-maze **(B)**. During Y maze testing, HPC theta power is reduced and HPC gamma power is elevated in the FKO mice **(C,D)**. HPC theta-gamma coupling was decreased in FKO mice **(E)**. No significant differences were observed in PFC power between WT and FKO mice **(F,G)**. Statistical significance denoted as *p < 0.05. MI, modulation index. pHPC, HPC phase. aPFC, PFC amplitude. pPFC, PFC phase. aHPC, HPC amplitude.

In addition to spontaneous oscillations, we examined task-related oscillations during Y-maze testing, as this might provide insight into the behavioral abnormalities and cognitive deficits exhibited by FKO mice. The spontaneous alternation Y-maze, which exploits the natural tendency of mice to explore novel environments, assesses spatial reference memory performance as mice make correct or incorrect alternations among the three maze arms (Kraeuter et al., 2019). We previously reported that FKO mice did not perform above chance level in this task, indicative of memory impairments (Foote et al., 2015). Similar to our prior results, WT mice had a significantly higher alternation percentage than FKO mice (*p* = 0.0348, Figure 3A). There was no statistically significant difference between WT and FKO mice in the number of maze arm entries (Figure 3B). By identifying differences in neural oscillations between FKO and WT mice as mice navigated the maze, we sought to correlate the memory deficit in FKO mice with changes in neuronal network activity.

Indeed, we observed statistically significant differences in band power and phase-amplitude coupling between 14-3-3 FKO and WT mice during the Y-maze. Similar to our resting-state results, decreased theta power in the HPC (*p* = 4.09e-4, Figure 3C,D) as well as decreased HPC theta-gamma coupling (*p* = 0.0060, Figure 3E) were observed in FKO mice during the Y-maze. Again, there were no significant differences in PFC neural oscillations between WT and 14-3-3 FKO mice (Figures 3F,G), showing that the aberrant network activity in the FKO mice is primarily localized to the HPC and not the PFC. However, during the Y-maze test, no group differences in coherence were observed (data not shown), and 14-3-3 FKO mice exhibited increased gamma power in HPC (*p* = 0.0115, Figures 3C,D). As increased HPC gamma power was unique to mice navigating the maze, this suggests that abnormal gamma activity may contribute to the spatial reference memory deficits exhibited by FKO mice.

### Effects of Region-Specific 14-3-3 Inhibition on WT Neural Oscillations

While the 14-3-3 FKO mouse line has high expression of difopein in the PFC and HPC, expression is not limited to these two regions. The precise contributions of the PFC, HPC, and other brain regions to the observed electrophysiological and behavioral defects remain unknown. We have previously demonstrated that viral-mediated inhibition is an effective strategy for probing region-specific contributions of 14-3-3 proteins in the brain, as 14-3-3 inhibition in the PFC and HPC together or HPC alone via this method induced psychomotor behaviors in adult WT mice (Graham et al., 2019). Here we used this same approach to examine the region-specific effects of 14-3-3 protein inhibition on neural oscillations.

**FIGURE 4:**
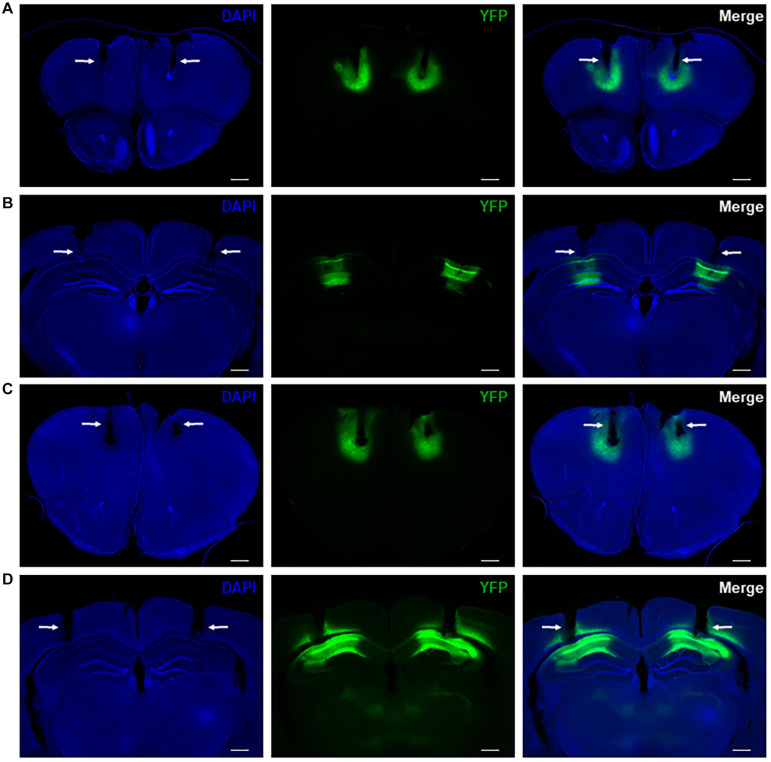
Representative histology showing bilateral virus expression and electrode tracks in mice with AAV-YFP injection in the PFC **(A)** and HPC **(B)** as well as mice with AAV-YFP-difopein injection in the PFC **(C)** and HPC **(D)**. Electrode tracks are indicated by white arrows. Virus expression is limited to a small region around the injection sites and is colocalized with recording sites. Scale bar = 500 μm.

We drove difopein expression in either the HPC or PFC of adult WT mice using an AAV vector that produces viral particles expressing YFP-difopein under the control of a CaMKIIα promoter (AAV-YFP-difopein). A separate virus lacking the difopein sequence was used as a negative control (AAV-YFP). Correct targeting of injections was confirmed via histology at the end of the experiment (Figures 4A–D). As previously reported (Graham et al., 2019), injection of AAV-YFP-difopein in the HPC but not PFC induced locomotor hyperactivity in the open field test (*p* = 0.0081, Supplementary Figure 3). Significant differences were observed between neural oscillations from the bilaterally implanted electrodes (*p* < 0.05 for 8 of the 10 measurements). Though we attempted to control for virus expression levels by excluding mice with asymmetrical YFP intensity from analysis, we cannot rule out the possibility that these differences are spurious. Nonetheless, data from each electrode was analyzed separately for the following experiments.

#### Resting-State Oscillations

**FIGURE 5:**
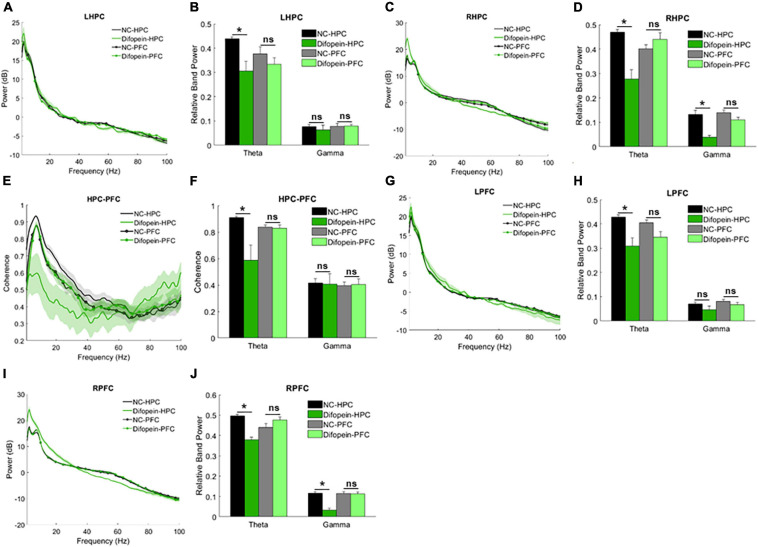
Resting-state power and coherence in WT mice injected with AAV-YFP negative control virus (NC-HPC, *n* = 7; NC-PFC, *n* = 10) and AAV-YFP-difopein (Difopein-HPC, *n* = 5; Difopein-PFC, *n* = 7). Difopein-HPC but not Difopein-PFC mice display decreased LHPC theta power **(A,B)**, decreased RHPC theta and gamma power **(C,D)**, decreased theta coherence between the HPC and PFC **(E,F)**, decreased LPFC theta power **(G,H)**, and decreased RPFC theta and gamma power **(I,J)**. Statistical significance denoted as *p < 0.05. *ns*, not significant.

WT mice with HPC injection of AAV-YFP-difopein (Difopein-HPC) exhibited decreased resting theta power in the HPC (*p*_left_ = 0.0121, *p*_right_ = 5.64e-5, Figures 5A–D) and decreased theta coherence between the HPC and PFC (*p* = 3.46e-5, Figures 5E,F) compared to AAV-YFP-injected mice (NC-HPC). While these results mirror those of 14-3-3 FKO mice, Difopein-HPC mice also exhibited a unilateral decrease in HPC gamma power (*p*_right_ = 1.74e-4, Figures 5A–D), an effect that was not observed in the FKO mice. There were no significant differences in neural oscillations between mice that received PFC injection of AAV-YFP-difopein and AAV-YFP (Difopein-PFC and NC-PFC, Figure 5). Moreover, AAV-YFP-difopein injection in either brain region did not induce any statistically significant differences in phase-amplitude coupling (data not shown).

Of note, Difopein-HPC mice also displayed decreased resting theta power (*p*_left_ = 0.0021, *p*_right_ = 0.0018) and unilaterally decreased gamma power (*p*_right_ = 8.81e-5, Figures 5G–J) in the PFC. Thus, 14-3-3 dysfunction in the HPC also alters neural oscillations in the PFC, where endogenous 14-3-3 activity is preserved. Just as AAV-YFP-difopein injection in the HPC, but not the PFC, is sufficient to induce psychomotor behaviors in WT mice (Graham et al., 2019), AAV-YFP-difopein injection in the HPC alone is able to affect neural network activity across both regions. Taken together, these results suggest that 14-3-3 dysfunction in the HPC is a larger contributing factor to the expression of schizophrenia-associated endophenotypes in FKO mice than 14-3-3 dysfunction in the PFC.

#### Task-Related Oscillations

**FIGURE 6:**
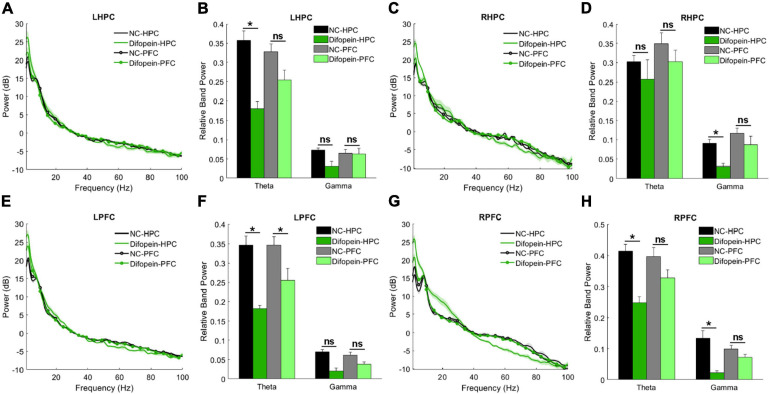
Task-related power in WT mice injected with AAV-YFP negative control virus (NC-HPC, *n* = 6; NC-PFC, *n* = 10) and AAV-YFP-difopein (Difopein-HPC, *n* = 5; Difopein-PFC, *n* = 7). LHPC theta power **(A,B)** and RHPC gamma power **(C,D)** are significantly decreased in Difopein-HPC but not Difopein-PFC mice. LPFC theta power is significantly decreased in both Difopein-HPC and Difopein-PFC mice **(E,F)**. RPFC theta and gamma power is significantly decreased in Difopein-HPC but not Difopein-PFC mice **(G,H)**. Statistical significance denoted as *p < 0.05. *ns*, not significant.

During the Y-maze, Difopein-HPC mice exhibited decreased theta power unilaterally in the HPC (*p*_left_ = 2.01e-4, Figures 6A–D) and bilaterally in the PFC (*p*_left_ = 0.024, *p*_right_ = 0.0092, Figures 6E–H) compared to NC-HPC mice. In addition, these mice had unilaterally decreased gamma power in both the HPC (*p*_right_ = 0.0484, Figures 6A–D) and PFC (*p*_right_ = 0.0011, Figures 6E–H). This is in direct contrast with 14-3-3 FKO mice, in which we observed elevated HPC gamma power during the Y maze test (Figures 3B,C). Difopein-PFC mice only showed a significant unilateral reduction in PFC theta power (*p*_left_ = 0.0415, Figures 6E,F), while every other measurement was not statistically different from that of NC-PFC mice. Moreover, there were no significant differences in coherence or theta-gamma coupling among groups during this task (data not shown).

**FIGURE 7:**
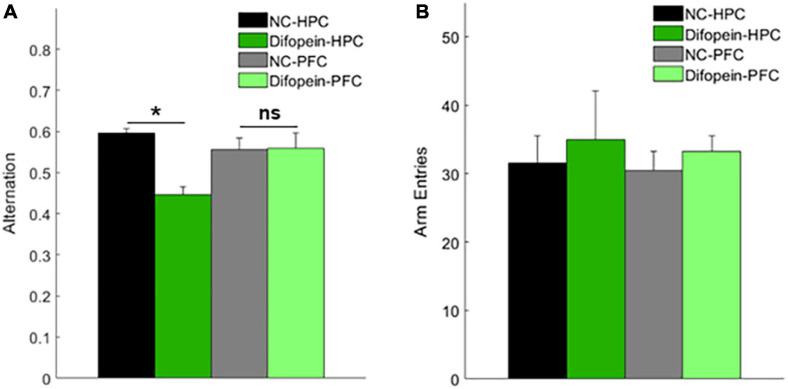
Mice with HPC AAV-YFP-difopein injection (Difopein-HPC, *n* = 5) alternate significantly less than any other group (NC-HPC, *n* = 6; NC-PFC, *n* = 10; Difopein-PFC, *n* = 8) in the spontaneous Y-maze **(A)**, indicative of spatial reference memory impairment. There are no significant differences among groups in the number of maze arm entries **(B)**. Statistical significance denoted as *p < 0.05. *ns*, not significant.

These results further demonstrate that acute viral-mediated 14-3-3 protein inhibition disrupts neural oscillations in the HPC and PFC, though the HPC seems to play a greater role in the expression of 14-3-3 dysfunction-induced abnormalities. Consistent with this, we found that Difopein-HPC mice perform worse than NC-HPC mice in the Y-maze (*p* = 6.55e-5, Figures 7A,B), while difopein injection in the PFC does not affect performance. The reversal of gamma power changes during the Y-maze in virus-injected groups compared to our prior experiment with WT and FKO mice suggests that acute, targeted 14-3-3 dysfunction and widespread, chronic 14-3-3 dysfunction have distinct effects on neural oscillations in mice. Whereas our two experimental models have opposing gamma power phenotypes, reduced theta power is a consistent indicator of cognitive impairment in mice with neuronal 14-3-3 protein inhibition.

## Discussion

The results of this study help to further elucidate the important role of 14-3-3 proteins in the mammalian brain. In particular, we found that resting-state and task-related theta and gamma oscillations in mice with acute and chronic 14-3-3 inhibition were altered in numerous ways, including abnormalities in LFP band power, coherence, and phase-amplitude coupling (Table 1). Aberrant theta oscillations were the most consistent feature across our two mouse models, perhaps highlighting the critical importance of 14-3-3 function to coordinated low-frequency network activity. As difopein expression in our mice is primarily limited to excitatory neurons (Qiao et al., 2014; Graham et al., 2019), this is in line with evidence suggesting that theta activity is regulated by interactions between hippocampal and medial PFC pyramidal cells (Colgin, 2011). At rest, FKO mice exhibited decreased HPC theta power, decreased HPC-PFC theta coherence, and decreased theta-gamma coupling within the HPC and between the PFC and HPC. While FKO mice in the Y-maze similarly exhibited decreased HPC theta power and decreased HPC theta-gamma coupling, they showed increased HPC gamma power as well. This suggests that abnormally elevated HPC gamma oscillations may play a role specifically in the spatial reference memory deficit observed in FKO mice.

**TABLE 1:** Summary of neural oscillation changes in acute and chronic 14-3-3 inhibition models.

	**FKO (vs WT)**	**HPC-Difopein (vs HPC-NC)**	**PFC-Difopein (vs PFC-NC)**
Resting band power	↓ HPC θ	↓ HPC θ (L/R), ↓ HPC γ (R), ↓ PFC θ (L/R), ↓ PFC γ (R)	No change
Resting HPC-PFC coherence	↓θ	↓θ	No change
Resting θ-γ coupling	↓ HPC, ↓ pPFC-aHPC	No change	No change
Maze band power	↓ HPC θ, ↑ HPC γ	↓ HPC θ (L), ↓ HPC γ (R), ↓ PFC θ (L/R), ↓ PFC γ (R)	↓ PFC θ (L)
Maze HPC-PFC coherence	No change	No change	No change
Maze θ-γ coupling	↓ HPC	No change	No change

*For virus models, the hemisphere(s) where significant differences were observed are indicated (left, right, or both). pPFC, PFC phase. aHPC, HPC amplitude.*

Similar to FKO mice, WT mice with virally induced 14-3-3 protein inhibition in the HPC displayed locomotor hyperactivity, decreased HPC theta power and HPC-PFC coherence at rest, decreased Y-maze performance, and decreased HPC theta power while navigating the maze. Acute viral 14-3-3 inhibition in the HPC was sufficient to induce neural oscillation defects not only in the HPC but also in the PFC where endogenous 14-3-3 activity is intact, suggesting that 14-3-3 dysfunction in the HPC has a pronounced effect on neural oscillations both locally and in distant brain regions. Unique perturbations in these mice include decreased HPC and PFC gamma power and decreased PFC theta power both at rest and during the Y-maze. Among the several possible explanations for the differences observed between the two mouse models, one is that a compensatory mechanism may alter neuronal network activity in the transgenic mice that express difopein since the perinatal period and thus develop largely without 14-3-3 protein activity in key brain regions. Another possibility is that difopein expression in brain regions outside the HPC and PFC of the FKO mice is responsible for the observed differences. Finally, differences in relative difopein expression levels between transgenic and virus-injected mice may play a role (Supplementary Figure 4). Also unique to our virus-injected mice is the observed asymmetry of neural oscillation changes. The differences in left and right hemisphere LFPs might reflect a biologically interesting phenomenon, but they also might simply be the result of unequal viral expression and stochasticity. More research on these topics is warranted, but our results lead to the conclusion that 14-3-3 inhibition, especially in the HPC, largely disrupts theta oscillations.

Theta oscillations are generally thought to coordinate the activity of widespread neuronal networks, and theta rhythms in the HPC and PFC are important for hippocampal-dependent memory functions, decision-making, and semantic processing (Buzsaki and Draguhn, 2004; Sigurdsson et al., 2010; Lisman and Jensen, 2013; Preston and Eichenbaum, 2013; Colgin, 2015; Shin and Jadhav, 2016; Barr et al., 2017). Patients with schizophrenia exhibit reduced theta oscillatory power during working memory tasks, and hippocampal-PFC theta synchrony is impaired in other animal models of schizophrenia such as in the mouse homolog to the 22q11.2 microdeletion syndrome model (Tamura et al., 2016). Gamma oscillations mediate a variety of cognitive processes including attention, sensory perception, sensorimotor integration, associative learning, and social cognition, all of which are impaired in schizophrenia (Moran and Hong, 2011; Uhlhaas and Singer, 2015; Sohal, 2016; Hunt et al., 2017). Abnormal resting-state gamma oscillatory power has been linked to schizophrenia pathology in numerous studies, and this phenomenon is hypothesized to underlie many of the behavioral and cognitive abnormalities exhibited by schizophrenic individuals (Basar-Eroglu et al., 2007; Spencer, 2011; Sun et al., 2011; Gandal et al., 2012; McNally and McCarley, 2016; Grent-’t-Jong et al., 2018). Here we studied theta and gamma network activity in the context of a family of genes that are known risk factors for schizophrenia. A greater understanding of how changes at the molecular, cellular, and circuit levels manifest as behavioral endophenotypes of schizophrenia may provide novel insights into the etiology and treatment of this highly intractable disorder.

These results, along with our previous findings (Foote et al., 2015; Graham et al., 2019), demonstrate that 14-3-3 protein dysfunction leads to synaptic, cellular, circuit, and behavioral defects in mice. Further research is needed to explore the differences between acute and chronic 14-3-3 inhibition and the inter-hemispheric differences in virus-injected mice. It may also be valuable to examine how neural oscillations in other brain regions are affected by 14-3-3 inhibition in the HPC and to determine the precise contributions of individual 14-3-3 isoforms to the observed results via isoform-specific genetic and viral-mediated 14-3-3 dysfunction. Finally, as gamma network activity arises from interactions among inhibitory interneurons (Sohal, 2016), manipulating 14-3-3 protein activity specifically in those neurons may provide further insight into the role of 14-3-3 proteins in gamma oscillations.

## Data Availability Statement

The raw data supporting the conclusions of this article will be made available by the authors, without undue reservation.

## Ethics Statement

The animal study was reviewed and approved by the Florida State University Animal Care and Use Committee.

## Author Contributions

ZBJ and YZ designed research. ZBJ performed experiments and analyzed data. JZ and YW assisted in collecting behavioral and imaging data. ZBJ and YZ wrote the manuscript. All authors contributed to the article and approved the submitted version.

## Conflict of Interest

The authors declare that the research was conducted in the absence of any commercial or financial relationships that could be construed as a potential conflict of interest.
