# Supplementary material for: Inhibition of 14-3-3 Proteins Alters Neural Oscillations in Mice
Source: Front Neural Circuits. 2021 Mar 12;15:647856. doi: 10.3389/fncir.2021.647856 (PMC7994333; doi:10.3389/fncir.2021.647856)
Supplement: Supplementary file 1 [file Data_Sheet_1.docx]

Supplementary Material

# Supplementary Figures and Tables

## Supplementary Figures


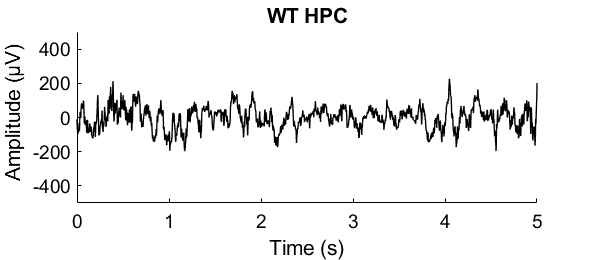


**A**


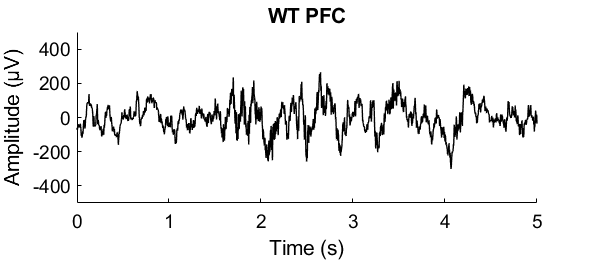


**B**


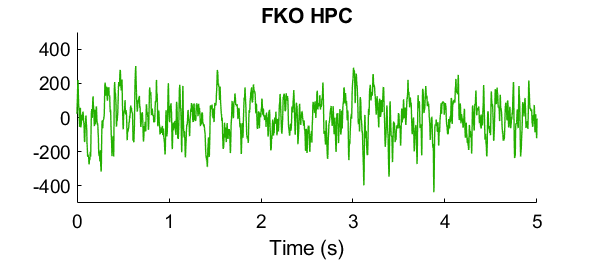


**C**


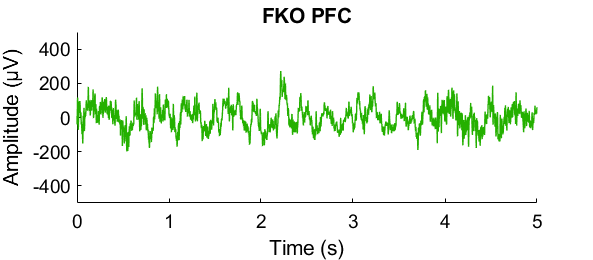


**D**


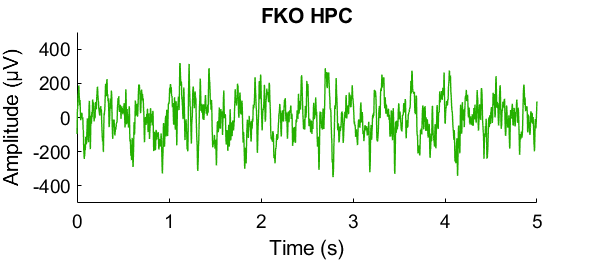


**Supplementary Figure 1.** Representative resting-state hippocampus and prefrontal cortex LFP traces from WT (A-B) and 14-3-3 FKO mice (C-D). Recordings are from chronic implanted electrodes sampled at a rate of 1 kHz with 1-100 Hz bandpass and 60 Hz notch filtering.


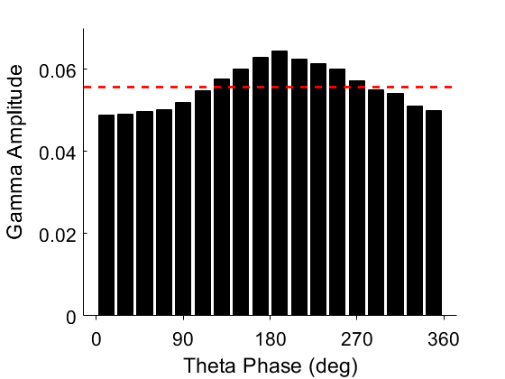

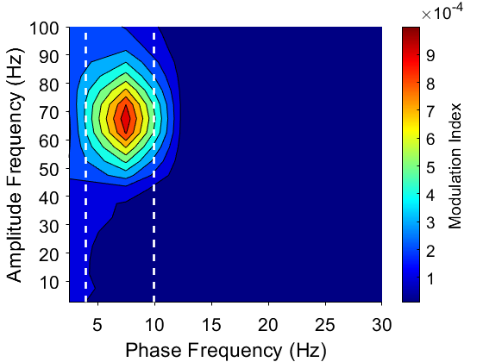

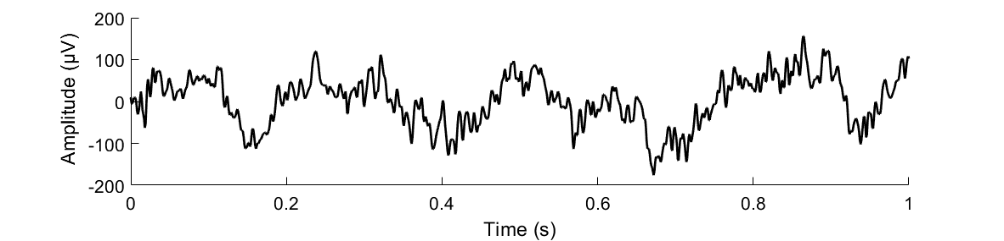


**A**

**B**

**C**


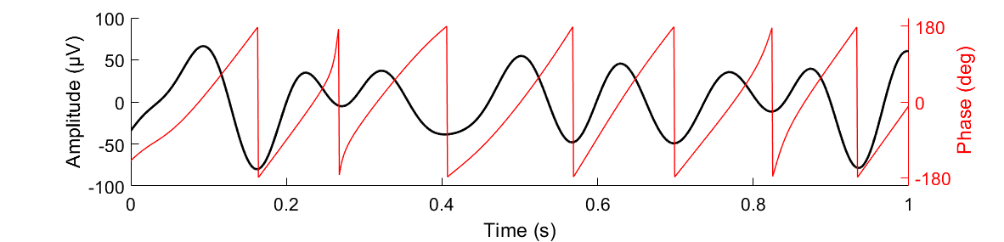


**Theta 4-10 Hz**


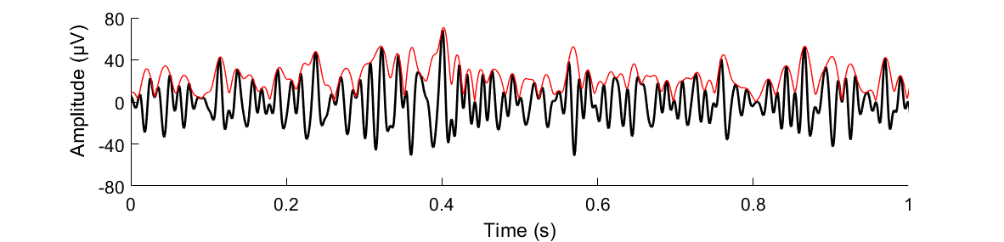


**Gamma 30-100 Hz**

**Supplementary Figure 2.** To quantify phase-amplitude coupling, the phase of the slower oscillation (A, middle) and the amplitude of the faster oscillation (A, bottom) are extracted from the LFP (A, top). Theta phase and gamma amplitude are computed from the Hilbert transform after filtering in the 4-10 Hz and 30-100 Hz frequency ranges, respectively. The distribution of mean gamma amplitudes is computed in 20° theta phase bins (B), and the divergence of this distribution from a uniform distribution (red line) is calculated as the modulation index. By following this procedure for multiple pairs of frequencies, the total phase-amplitude coupling can be quantified in a comodulogram (C). All data presented here were taken from representative WT HPC recordings, and the comodulogram shows strong coupling between theta phase (white lines) and gamma amplitude.


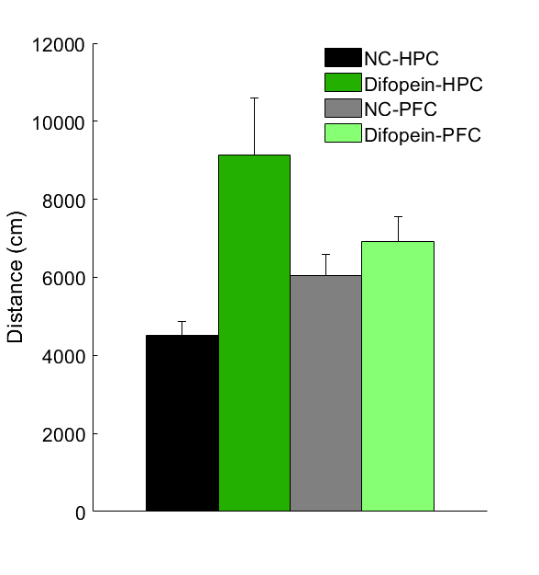


**ns**

*

**Supplementary Figure 3.** AAV-YFP-difopein expression in the HPC (Difopein-HPC, *n* = 5), but not PFC (Difopein-PFC, *n* = 8), induces locomotor hyperactivity in WT mice compared to mice injected with AAV-YFP negative control virus (NC-HPC, *n* = 6; NC-PFC, *n* = 10). Statistical significance denoted as **p* < 0.05. *ns* = not significant.

**Supplementary Figure 4.** YFP-difopein expression, estimated by fluorescence signal intensity, is higher in the PFC (**A**) and HPC (**B**) of WT virus-injected mice compared to transgenic FKO mice. 40 µm sagittal sections were imaged with identical exposure times on a Keyence BZ-X700 fluorescence microscope using a 20X objective. Scale bar = 100 µm.

**A**


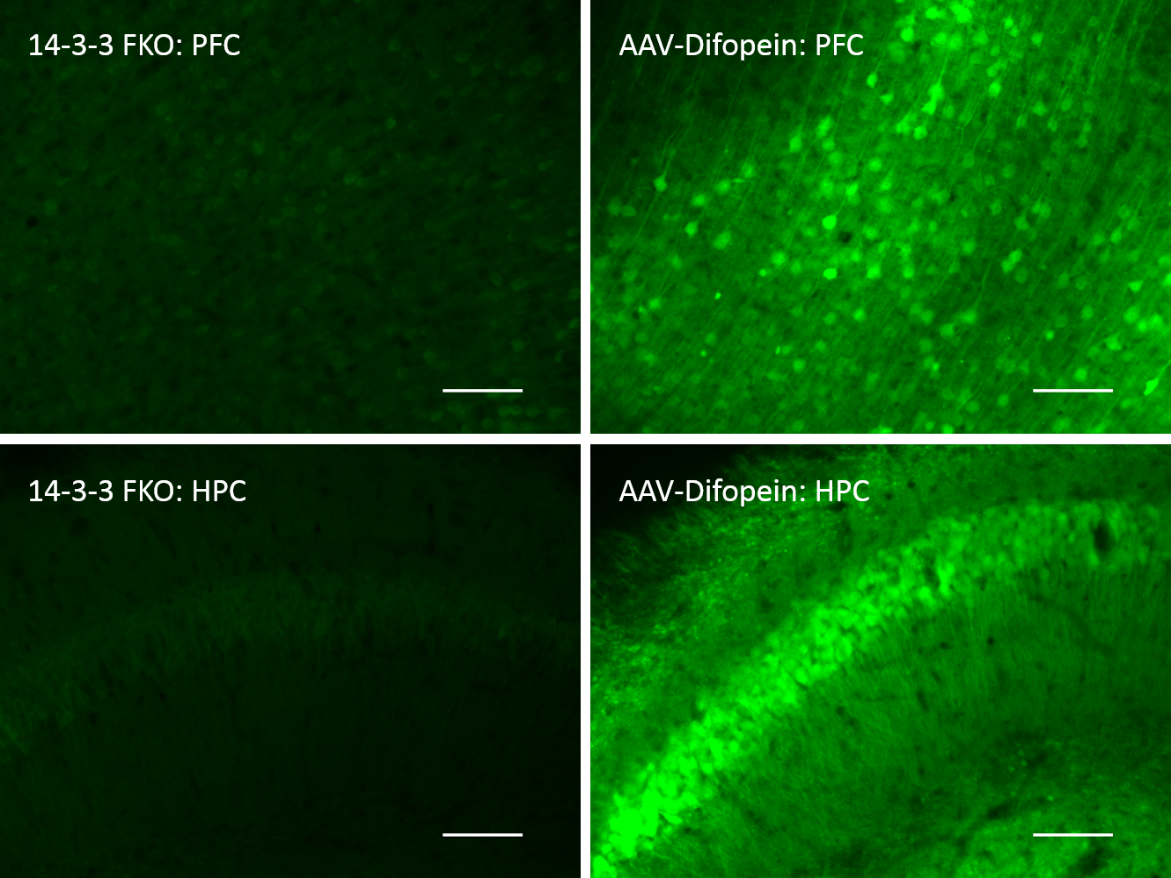


**B**

## Supplementary Tables

|  | WT Saline (*n* = 16) | WT Clz (*n* = 8) | WT Hal (*n* = 8) |
| --- | --- | --- | --- |
| HPC Theta Power | 0.362 ± 0.0219 | 0.395 ± 0.0299 | 0.392 ± 0.0285 |
| HPC Gamma Power | 0.0784 ± 0.00815 | 0.0704 ± 0.00866 | 0.0639 ± 0.0157 |
| PFC Theta Power | 0.334 ± 0.0146 | 0.371 ± 0.0230 | 0.374 ± 0.0208 |
| PFC Gamma Power | 0.0663 ± 0.00573 | 0.0555 ± 0.00361 | 0.0737 ± 0.0131 |
| HPC-PFC Theta Coherence | 0.465 ± 0.0417 | 0.383 ± 0.0447 | 0.439 ± 0.0911 |
| HPC-PFC Gamma Coherence | 0.316 ± 0.0384 | 0.211 ± 0.0268 | 0.349 ± 0.0819 |
| HPC TG Coupling | 5.42e-04 ± 1.15e-04 | 4.71e-04 ± 1.16e-04 | 5.02e-04 ± 1.53e-04 |
| PFC TG Coupling | 1.03e-04 ± 2.63e-05 | 1.54e-04 ± 4.74e-05 | 5.50e-05 ± 1.05e-05 |
| pHPC-aPFC TG Coupling | 5.96e-05 ± 1.17e-05 | 5.90e-05 ± 1.38e-05 | 3.50e-05 ± 2.26e-05 |
| pPFC-aHPC TG Coupling | 2.50e-04 ± 7.27e-05 | 2.48e-04 ± 7.32e-05 | 2.76e-04 ± 1.01e-04 |

**Supplementary Table 1.** Antipsychotic drug administration does not alter neural oscillations in WT mice. Clz = clozapine (2 mg/kg). Hal = haloperidol (0.4 mg/kg). pHPC = HPC phase. aPFC = PFC amplitude. pPFC = PFC phase. aHPC = HPC amplitude. Data are presented as mean ± standard error of the mean. *p* > 0.05 for each measurement (1-way ANOVA).

|  | FKO Saline (*n* = 18) | FKO Clz (*n* = 10) | FKO Hal (*n* = 9) |
| --- | --- | --- | --- |
| HPC Theta Power | 0.312 ± 0.0156 | 0.340 ± 0.0278 | 0.393 ± 0.0200 |
| HPC Gamma Power | 0.116 ± 0.0130 | 0.0835 ± 0.0153 | 0.0766 ± 0.00439 |
| PFC Theta Power | 0.340 ± 0.0139 | 0.361 ± 0.0182 | 0.402 ± 0.0160 |
| PFC Gamma Power | 0.0835 ± 0.00939 | 0.0644 ± 0.00905 | 0.0596 ± 0.00509 |
| HPC-PFC Theta Coherence | 0.409 ± 0.0299 | 0.418 ± 0.0585 | 0.324 ± 0.0335 |
| HPC-PFC Gamma Coherence | 0.333 ± 0.0285 | 0.347 ± 0.0414 | 0.239 ± 0.0248 |
| HPC TG Coupling | 1.39e-04 ± 2.97e-05 | 1.61e-04 ± 2.07e-05 | 1.21e-04 ± 2.41e-05 |
| PFC TG Coupling | 8.90e-05 ± 2.33e-05 | 8.17e-05 ± 1.30e-05 | 7.07e-05 ± 2.24e-05 |
| pHPC-aPFC TG Coupling | 6.11e-05 ± 1.17e-05 | 9.59e-05 ± 2.59e-05 | 5.53e-05 ± 1.34e-05 |
| pPFC-aHPC TG Coupling | 7.85e-05 ± 1.47e-05 | 7.10e-05 ± 1.02e-05 | 8.97e-05 ± 2.55e-05 |

**Supplementary Table 2.** Antipsychotic drug administration does not alter neural oscillations in FKO mice. Clz = clozapine (2 mg/kg). Hal = haloperidol (0.4 mg/kg). pHPC = HPC phase. aPFC = PFC amplitude. pPFC = PFC phase. aHPC = HPC amplitude. Data are presented as mean ± standard error of the mean. *p* > 0.05 for each measurement (1-way ANOVA).
